# Review the factors associated with dietary sodium adherence in patients with heart failure from selected research-based literatures

**DOI:** 10.1186/s40795-022-00536-5

**Published:** 2022-05-03

**Authors:** Yi-Wen Lee, Chien-Ning Tseng

**Affiliations:** 1 College of Nursing, Chang Gung University of Science and Technology, No 261, Wen-Hwa 1st Rd., Kwei-Shan, Taoyuan City, 33303 Taiwan, Republic of China; 2 Department of Nursing, Asia Eastern University of Science and Technology, No. 58, Sec. 2, Sichuan Rd., Banqiao Dist., New Taipei City, 220 Taiwan, Republic of China

**Keywords:** Adherence, Dietary sodium, Heart failure

## Abstract

**Background:**

Low-sodium dietary is an important measure to avoid heart failure patient’s body fluid volume overload. There are still more than 50% of heart failure patients who are incapable of complying low-sodium dietary. This study utilized the systematic literature review method, with the purpose to understand the related factors towards the compliance behavior of low-sodium dietary of patients with heart failure.

**Methods:**

This study typed keywords (congestive heart failure, adherence, compliance, low sodium diet, low salt diet, dietary sodium restriction) from computer databases (CINAHL, Cochrane, Medline, ProQuest Nursing Allied Health, PubMed, and ScienceDirect) and according to inclusion criteria were as follows: (1) Research objects were adult patients admitted to HF, (2) Research content were related to sodium diet compliance behavior. (3) Non-intervention research. Exclusion criteria were as follows: (1) participants who was pregnant women, (2) qualitative research, (3) studies on development of tool, (4) poster. Select needed 14 articles that meet the purposes of this study. In addition, the study also collected 4 more studies with the same purpose from the references that were included in the retrieved articles, and finally a total of 18 studies were included in the analysis of this study. Data analysis uses descriptive statistics (percentage, frequency distribution) and content analysis method.

**Results:**

This study use a systematic literature review method, it was found that the results of the studies use nonintervention method, which explored the relevant factors of low-sodium dietary compliance in heart failure patients over the past 20 years, could be summarized in 4 dimensions as social and economic conditions, patient conditions, disease conditions and therapy conditions.

**Conclusions:**

Heart failure patients’ compliance behavior toward low-sodium dietary is multi-dimensional. Therefore, this study recommends when the health care providers evaluating patients’ compliance behavior of low-sodium dietary, it is necessary to consider more different aspects.

## Introduction

Heart failure (HF) is an important health-related issue because of the large population and the high cost of medical care nowadays. It was estimated that there were about 26 million people admitted to HF globally [1] and the United States will spend 2.5 times as much on HF expense by 2030 as it did in 2012 (20.9 billion in 2012; 53.1 billion in 2030) [2].

Symptoms of fluid volume overload for HF are common and serious. According to Kammar-García et al. (2018), studies had shown that 71.6% of patients with HF who receive treatment in emergency department also had symptoms of fluid volume overload. So it is critical to monitor the patients’ body fluid volume retention, because it strongly predicts the readmission and the event-survival time [3].

The cause of physiological mechanism of the fluid volume overload is not yet known currently. Some scholars believed that body fluid volume overload may be related to the long-term activation of the neuro-hormonal system and interference of constant physiological body fluids [4], while others believed that fluid volume overload positively correlates with the sodium retention in the body [5]. Patients’ excessive body fluid volume state would benefit from low-sodium diet which was mostly recommended by health care workers reasons were: (1) High-sodium diet will change the various properties of proteins that responsible for calcium balance and myocardial contractility, which leads to the cause of myocardial constriction, development of hypertension and various cardiovascular complications [6]. (2) If patients with hypertension continued the adoption of high-sodium diet, it will increase the risk of cardiovascular events and death rate [7]. Hypertension is also one of the causes of HF. (3) The higher the sodium intake, the higher the risk of acute decompensated heart failure [8]. (4) Patients admitted with HF symptoms accompanied by low-sodium diet performed better in prognostic clinical prediction [9]. (5) Sodium intake below 3 g per day could positively improve symptom burden and clinical health outcome in patients with HF [10].

Many scholars and experts [11, 12] believed that attention to patient sodium intake was the focus of care for HF. But according to the result of systematic literature review process, 50–88% of HF patients failed to follow the recommendations of a low-sodium diet [13]. HF is a chronic disease, theoretically patients should have multiple opportunities to receive dietary care, but many of them were still unable to follow the recommendations of a low-sodium diet. Therefore, this study used the systematic literature review method to understand the factors of patients’ low-sodium dietary compliance behavior. It is expected that this research result will assist to improve the care for patients with HF who comply with low-sodium diet.

## Methods

**Fig. 1:**
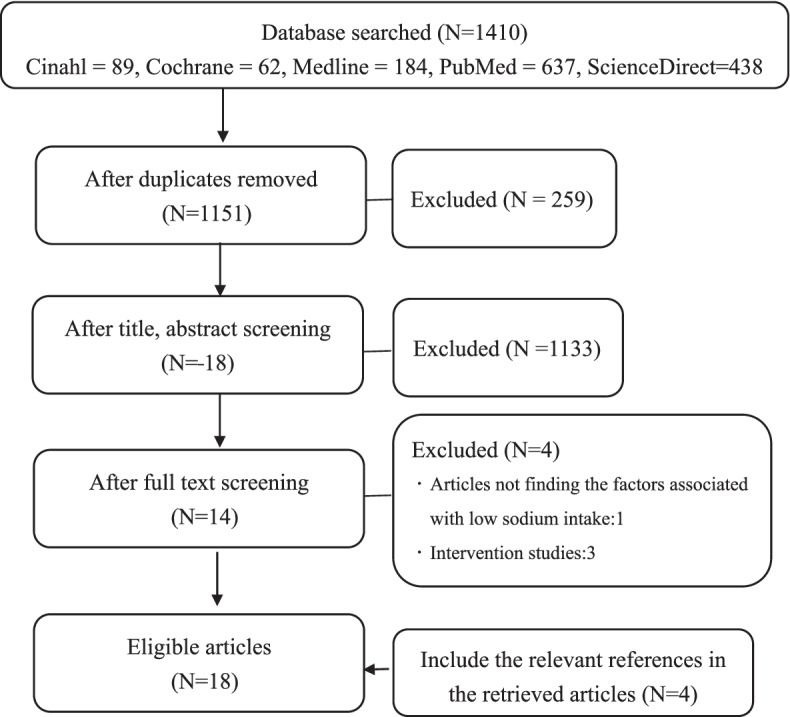
Systematic review flowchart

This study collected non-interventional research literatures that concerned the low-sodium diet behavior of the patients with HF by systematic literature review method, further to understand the relevant factors associated with. The included literatures in the analysis were collected in a stepwise process, keywords were typed (congestive heart failure, heart failure, adherence, compliance, low-sodium diet, low salt diet, dietary sodium restriction) from computer databases (CINAHL, Cochrane, Medline, ProQuest Nursing Allied Health, and PubMed, and ScienceDirect), and searched the database for English literature from insight to 2020, a total of 1410 articles gained, there were 259 duplicates removed. The rest of 1151 articles were learned from which title and abstract sorted by inclusion and exclusion criteria, again 1133 were deleted afterwards. Three intervention studies were deleted from the remained 18 full articles, one article that was not finding the factors associated with low sodium intake were removed further, and finally selected the 14 articles that meet the purposes of this study. In addition, this study also collected 4 more studies with the same purpose from the relevant references section of the retrieved articles, and eventually a total of 18 studies were included in the analysis of this study (Fig. 1).

The conditions to include the articles in the analysis were: (1) Research objects were adult patients admitted to HF. (2) Research content were related to sodium diet compliance behavior. (3) Non-intervention research. Exclusion criteria were as follows: (1) participants were pregnant women, (2) qualitative research, (3) studies on development of tool, (4) poster.

### Analysis

Data Analysis was computed and processed by descriptive statistics and content analysis. Descriptive statistics were mainly the presentation and application of percentage, frequency distribution, characteristics description of this study. Whilst content analysis was mainly the analysis based on HF patients who comply with the behavior of low-sodium diet.

Five procedures of content analysis were: (1) Collection and organization of all information included in the retrieved articles: review the integrity of data collection to number and file them in well-ordered manner. (2) Familiarization of analysis yet to be completed: the researchers read all the data repeatedly to familiarize themselves with the nature of the data and the ideas generated by notes and annotations taken. (3) Identification of the unit of analysis: the purpose of the study was followed by the determination of unit of analysis as the researchers took the factors of compliance behavior on restricted sodium diet. (4) Numbering and coding the research results that were included in analysis in a proper-order manner. (5) Generalization of the meaning unit into categories [14, 15]. The classification framework of this study referred to the 5 dimensions of medication instruction adherence behavior that were proposed by World Health Organization (WHO) (2003) [16]. The rigorous approach to data analysis was the re-examination of the categorized data after two to 4 weeks of interval and consulted other researchers to review all the analytical and categorized data.

## Results

### The characteristics of the analyzed article

There were 19 articles included in the analysis which was published between the year 2001–2020 (*n* = 19), nine articles as secondary analysis [17–25], one article in pilot study (longitudinal design) [26], eight articles in original study (cross-sectional design) [27–34]. Except one of the articles published from South Korea [29], the rest were from the United State (*n* = 17) [17–28, 30–34]. As in study population, except one article paired patients with HF (mostly male) and primary care providers (mostly female), there were 114 patients recruited as research subjects [20], while the remaining research subjects were received as outpatients (*n* = 14) [17–19, 21–27, 29, 31–33], male (*n* = 17) mostly [17–19, 21–34], number of recruitment cases between 24 and 445 [17–19, 21–34], with an average age of 54–71 years [17–19, 21–34]. In terms of patients’ HF characteristics, the mean Left Ventricular Ejection Fraction (LVEF) was between 27 and 68% [17, 20–30, 32–34]. New York Heart Association (NYHA) Functional Classification (FC) was dominated by I ~ II mostly (*n* = 9) [17, 18, 20, 21, 27, 29, 31, 33, 34]. In the method of sodium intake estimation, self-reports were majorly used (e.g. diet logs, questionnaires, interviews, checklists, etc.) (*n* = 7) [18, 19, 26, 29–31, 34], followed by urinary sodium excretion (*n* = 8) [21–25, 28, 32, 33]. The reports of sodium intake estimation that utilized both urinary sodium excretion and self-reports simultaneously were least (*n* = 3) [17, 20, 27] (Table 1).

**Table 1:** Characteristic of included studies

Source	Country	Study type and designs	Population	Sample size	Age, Years	Men gender, %	NYHA FC, %	Mean LVEF, %	Method to estimate the sodium intake
Lennie et al., 2020 [17]	United States	Secondary analysis	Outpatient	223	62 ± 12	59	I-II: 54.3- III-IV:45.7	36	UNa, Self-report
Smith et al., 2019 [18]	United States	Secondary analysis	Outpatient	255	61 ± 12	68	I-II: 56.1 III-IV:43.9	No data found	Self-report
Sattler et al., 2019 [19]	United States	Secondary analysis	Outpatient	445	71 ± 10	54	No Data Found	No data found	Self-report
Bidwell et al., 2018 [20]	United States	Secondary analysis	Outpatient	114 dyads (P-C D)	56 ± 10	63	II:71.9 III: 28.1	27	UNa, Self-report
Wu et al., 2017 [21]	United States	Secondary analysis	Outpatient	244	62	67	I-II:53.5 III:32.9 IV:13.6	34	UNa
Basuray et al., 2015 [22]	United States	Secondary analysis	Outpatient	305	69 ± 10	64	I-II:39 III-IV:61	32	UNa
Chung et al., 2015 [23]	United States	Secondary analysis	Outpatient	379	62 ± 12	67	I-II:49.1 III:38.3 IV: 11.3	34	UNa
Masterson Creber et al., 2014 [24]	United States	Secondary analysis	Outpatient	280	62 ± 12	64	I-II:23 III:59 IV:18	35	UNa
Masterson Creber et al., 2014 [25]	United States	Secondary analysis	Outpatient	279	62 ± 12	64	I-II:23 III:59 IV:18	35	UNa
Riegel et al., 2019 [26]	United States	Pilot, Longitudinal	Outpatient	24	592 ± 92	67	II: 29.2 III:50 IV:20.8	68	Self-report
Chung et al., 2017 [27]	United States	Original, Cross-sectional	Outpatient	74	61 ± 14	59	I-II:77% III-IV:23%	39	UNa, Self-report
Dolansky et al., 2016 [28]	United States	Original, Cross-sectional	Outpatient Inpatient	339	69 ± 10	61	I: 10.3 II:24.2 III: 60.2 IV:5.3	30	UNa
Hwang et al., 2016 [29]	South Korea	Original, Cross-sectional	Outpatient	91	57 ± 14	67	I: 49.4 II:42.9 III: 5.5 IV: 2.2	53	Self-report
Alosco et al., 2013 [30]	United States	Original, Cross-sectional	Unclear	149	68 ± 11	63	II-III:100	41	Self-report
Luyster et al., 2009 [31]	United States	Original, Cross-sectional	Outpatient	88	70 ± 11	77	I:46 II: 52 III: 2	ICD	Self-report
Lennie et al., 2008 [32]	United States	Original, Cross-sectional	Outpatient	145	65 ± 14	55	II:44 III:47 IV:9	33	UNa
Chung et al., 2006 [33]	United States	Original, Cross-sectional	Outpatient	68	63 ± 14	60	I:5.9 II:45.6 III-IV:48.5	30	UNa
Evangelosta et al., 2001 [34]	United States	Original, Cross-sectional	Outpatient Inpatient	82	54 ± 13	62	I:16 II:42.7 III:35.4 IV:6	28	Self-report

*Abbreviations*: *NYHA FC* New York Heart Association Functional Classification, *LVEF* Left Ventricular Ejection Fraction, *P-C D* Patient- Caregiver Dyads, *UNa* Urinary sodium excretion, *ICD* Implantable Cardioverter Defibrillator

### Related factors of compliance behavior towards low-sodium diet

This study identified meaning units through content analysis which were classified into four categories (Table 2). The four categories were: social and economic conditions, patient conditions, disease conditions and therapy conditions. Social and economic conditions contained two subcategories: personal demographics and attribute information. Patient conditions include three subcategories: health, social support, and self-care.

**Table 2:** Related factors of compliance behavior towards low-sodium diet

Theme	Categories	Subcategories	Items
Dietary sodium adherence	Social and economic conditions	Personal demographics	Age; Gender; Race
		Attribute information	Education level; Income level; Marital status; Body mass index
	Patient conditions	Health	Cognitive function; Diabetes; Nutrition; Mental health; physical health
		Social support	The attitude of significant others (partner/mate) towards the low-sodium diet; Anyone who eat on the same table also followed a low-sodium diet; Whether there is a chef to prepare food at home.
		Self-care	Knowledge of low-sodium diet; Willpower; Motivation
	Disease conditions		Period of suffer from heart failure; Severity of heart failure
	Therapy conditions		Medication

#### Social and economic conditions

Ten included articles (55.56%) in this study demonstrated the low-sodium dietary compliance was associated with social and economic conditions. Social and economic conditions referred to two categories which were personal demographics and attribute information. Personal demographics subcategorized as age [25, 31], gender [17, 21, 29, 33], race [31]. Whereas attribute information subcategorized as education level [28], income level [28], marital status [31], body mass index [18, 22, 24, 25, 28].

#### Patient conditions

Thirteen included articles (72.22%) in this study showed that low-sodium dietary compliance was associated with patient conditions. Patient conditions contained three subcategories, health, social support, and self-care. Health subcategory consisted of cognitive function [24, 25], diabetes [24, 30], nutrition [19], mental health [29, 31, 34], physical health [21]. Social support subcategory referred to factors such as the meaningfulness and attitude of the significant others of the patients toward a low-sodium diet [21], anyone who eat together also followed a low-sodium diet [23], if there is a chef helps to prepare the food at home [28]. Self-care subcategory, such as patients’ knowledge to low-sodium diet [20, 28], patients controlled their impulsivity of eating high-sodium foods (willpower) [34], incentive to follow a sodium diet (motivation) [27, 32, 34].

#### Disease conditions

Disease conditions referred to the characteristics of the condition in patients with HF. Two included articles (11.11%) in this study showed that low-sodium dietary compliance was associated with disease conditions. For example: period of suffering from HF [26], severity of HF [21].

#### Therapy conditions

Therapy conditions referred to treatment related to HF. One included article (5.56%) in this study showed that low-sodium dietary compliance was associated with therapy conditions, such as the medication [18].

## Discussion and conclusions

Mattes (1990) divided the research subjects into 3 equivalent groups, different degrees of restriction on low-sodium diet were suggested respectively: highly strict restriction, discretionary restriction, and no restriction. The result merely indicated the unhelpfulness of execution of low-sodium diet by patients with HF disregarded the degrees of limitation suggested, which might even be counterproductive in the end. This result signified there should be other unnotified factors for enhancement of the patient’s compliance behavior of low-sodium diet [35]. The 18 non-interventional research literatures of this study collected by systematic literature review method had applied both qualitative analysis and the classification framework of the 5 dimensions of medication instruction adherence behavior proposed by WHO (2003).

Result classified the factors of adoption of low-sodium diet by patients with HF over the past 20 years into four dimensions: social and economic conditions, patient conditions, disease conditions and therapy conditions. Patients with HF tend to have low willingness to follow the low-sodium diet. According to the studies of Basuray et al., the rate of adoptability of low-sodium diet compliance of patients were merely 14–23% (*n* = 305). Clinically it was considered the visualization and the sensation of taste of the meal as the argument to patients’ low willingness [22].The result of this study was consistent with what WHO (2003) proposed: a compliance behavior is multi-dimensional factors [16]. Therefore, this study recommended that health caregivers should have a multi-dimensional consideration when evaluating patients’ low-sodium dietary behavior.

This study did not illustrate the correlation between health care system conditions and compliance behavior mentioned by WHO (2003). This might be the cause of limitation to the articles included in this study were majorly secondary analysis, or the limited generalizability of few articles/studies that discussed the correlation between health care system conditions and compliance behavior of low-sodium diet at presence. Health care system conditions contained HF care equipment, policy-related factors and care guideline. The care guideline from different societies existed inconsistencies, for instance, The European Society of Cardiology (ESC) is neutral about whether patients with HF followed a low-sodium diet [36]. The Heart Failure Society of America recommends a daily sodium intake of between 2000 and 3000 mg for mild patients and less than 2000 mg per day for moderate to severe patients [37]. The American College of Cardiology Foundation (ACCF) /American Heart Association (AHA) recommended classification by stages depending on the severity of HF patients**.** The consumption of patients in the stages A and stage B were less than 1500 mg of sodium per day, and those in the stages C and Stages D consumed less than 3000 mg of sodium per day [38]. If reliability and professionality of organizations as such do not share the common consistent point of view towards low-sodium diet related to HF, this might cause the health care providers offered the inconsistent information or instruction when health-educating the patients which furthermore affected the low-sodium compliance of patients. Hence it would certainly be beneficial from exploration of consistent recommendations to the amount of daily sodium intake of patients with HF.

According to Kuehneman et al., studies exhibited the decompensation patients’ capability of successful avoidance to the excessive ingestion of dietary sodium through food hygiene education and the tracking system regardless meal ordered from cafeteria within hospital or take out from restaurant during hospitalization. Thus, the healthcare equipment proved to possibly affect the low-sodium diet compliance behavior of patients [39]. For the purpose of enforcement of the low-sodium diet compliance behavior, the relationship between healthcare system and low-sodium diet worth further discussions.

The result of this study indicated that the major method of estimation of sodium intake was self-report, which followed by sodium urine excretion method. This result unlike Sousa et al. (2020) [40], whereas the literature which analyzed 13 documents and demonstrated that most literatures estimated sodium intake were using sodium urine excretion method. The possible reason of unlikeness might be the literatures included in the analysis.

It was essential to understand sodium intake when exploring compliance with a low-sodium diet. There were three broad ways to estimate the quantity of sodium intake: by sodium urine, by using a duplicated portion, by self-reporting (e.g. dietary questionnaires, dietary logs, dietary memory records, etc.) [41]. These ways of estimation helped to comprehend the amount of sodium intake, but there were some limitations to be aware of. For example, estimation process of sodium intake with sodium urine required a high degree of cooperation from the patient and the patient’s ability to collect urine in its entirety [28]. Using a duplicate portion to estimate sodium intake required the preparation of two exact servings of food, one for the patient’s diet and one for analysis of the sodium in the content of the food. Uneconomical feelings might occur in third person’s view (waste of food). Consultation to patients by questionnaire to self-confessed compliance with the sodium diet, it might not provide a true understanding of the patient’s sodium intake amount. By asking the patient either to make a dietary log or a dietary memory record for the estimation of sodium intake amount, it required the patient to scale the food weight before and after meals respectively. However, it does not meet with the people’s general eating habits. As well as the need of the patient capability to correctly use the scale, correctly recorded and recall the type of food eaten, quantity etc. In addition, the accessibility and availability of computer software assistance is to be considered, whether the computer software has the effect of ingredient analysis of homemade cultural food [41]. Thus, the finding of convenience and accuracy to the estimation of sodium intake amount is a necessary future effort.

Although this study presented multi-dimensional factors that related to patient’s low-sodium diet compliance behavior, the items in each dimension proposed were inconsistent with the relation to the behavior. For example, in the case of patient conditions, studies had shown that no significant difference in performance of low-sodium dietary compliance between patients with HF and patients suffered with both HF and diabetes (*p* = 0.810) [27]. But meanwhile there were also studies shown that the patients suffered with both HF and diabetes tended to have excessive sodium intake amount over the recommended amount (*p* = 0.016) [25]. Studies of Creber et al. [24]showed that patients with both HF and diabetes tended to have adoption of higher sodium diet [24]. In the case of therapy conditions, studies [25] had shown that patients who used diuretics cannot predict their low-sodium diet compliance behavior. There were literatures [9] also mentioned that the use of diuretics caused dehydration, less saliva discretion affected the speed of food breakdown, which in turn changed the feeling of taste and salt-taste sensitivity. The Angiotensin-Converting Enzyme Inhibitors (ACEI) remained not concluded. Studies [18] have revealed that patients who followed ACEI had 13% more sodium consumption than patients who did not. But also there were other studies [22] indicated ACEI did not cause differences in low-sodium diet compliance behavior. In the case of social and economic conditions, studies [33] indicated female patients had higher compliance behavior in comparison with male patients, which studies of Wu et al.(2017), also shown the result of higher dietary sodium intake amount of male patients (4.04 g) than female patients (3.35 g). Studies of Lennie et al. (2008) presented the gender difference in the appetite response due to basic energy intake and energy density requirement which males’ calorie intake amount were more than female’s intake amount generally. But sodium intake amount do not vary by gender difference even the involvement of the calories intake amount was taken into consideration [32]**.**

As in the disease conditions, though the compliance behavior of low-sodium diet was significantly related to the severity [21] and the suffering period of HF [26]. Since the literatures and articles included in the analysis of this study were not enough, further discussion is necessary in topics like the correlation of the variance curve of low-sodium diet compliance behavior associated with the movement in the timeline, as well as the severity of HF and compliant behavior of low-sodium diet.

This study believed if patients were unable to follow the health caregiver’s low-sodium dietary recommendations, and meals are essential for daily life, this advice should be stressful for those who fail to comply. Through this study, one could understand the factors related to patients’ low-sodium dietary compliance behavior, and hope it could be advantageous in improvement of patients’ compliance with low-sodium diet behavior.

## Limitations

Each literature has different importance of contribution to the academic system. Although the information used by secondary analysis usually encounter the question of credibility and validity, but discussion of the serious topic such as HF patients’ diet do require the consideration of manpower and material costs, secondary analysis can be a good approach in this case. Because of the above reasons, this research screened out a number of secondary analysis literatures after inclusion criteria.

This study described only 18 studies included in the analysis, and while these results could serve as reference for improvement of compliance with low-sodium diet behavior in patients with HF, it is critical to remind readers that this study did not cover all the potential factors related to patients’ low-sodium dietary compliance, still there was a need to provide more comprehensive and complete healthcare surroundings in terms of patient individuality.

## Data Availability

This was a systematic review. All of the included studies were elaborated in the finding.

## References

[1] Ambrosy AP, Fonarow GC, Butler J, Chioncel O, Greene SJ, Vaduganathan M (2014). The global health and economic burden of hospitalizations for heart failure: lessons learned from hospitalized heart failure registries. J Am Coll Cardiol.

[2] Hauptman PJ (2018). Global Health and heart failure. J of Card Fail.

[3] Kammar-García A, Pérez-Morales Z, Castillo-Martinez L, Villanueva-Juárez JL, Bernal-Ceballos F, Rocha-González HI (2018). Mortality in adult patients with fluid overload evaluated by BIVA upon admission to the emergency department. Postgrad Med J.

[4] Masella C, Viggiano D, Molfino I, Zacchia M, Capolongo G, Anastasio P, Simeoni M (2019). Diuretic resistance in cardio-nephrology: role of pharmacokinetics, Hypochloremia, and kidney remodeling. Kidney Blood Press Res.

[5] Bennett SJ, Huster GA, Baker SL, Milgrom LB, Kirchgassner A, Birt J (1998). Characterization of the precipitants of hospitalization for heart failure decompensation. Am J Crit Care.

[6] Patel Y, Joseph J (2020). Sodium intake and heart failure. Int J Mol Sci.

[7] Mente A, O'Donnell M, Rangarajan S, Dagenais G, Lear S, McQueen M (2016). Associations of urinary sodium excretion with cardiovascular events in individuals with and without hypertension: a pooled analysis of data from four studies. Lancet..

[8] Arcand J, Ivanov J, Sasson A, Floras V, Al-Hesayen A, Azevedo ER (2011). A high-sodium diet is associated with acute decompensated heart failure in ambulatory heart failure patients: a prospective follow-up study. Am J Clin Nutr.

[9] Sousa MM, Gouveia BLA, Almeida TCF, Freire MEM, Melo FABP, Oliveira SHDS (2020). Evidence related to sodium restriction in patients with heart failure. Rev Bras Enferm.

[10] Son YJ, Lee Y, Song EK (2011). Adherence to a sodium-restricted diet is associated with lower symptom burden and longer cardiac event-free survival in patients with heart failure. J Clin Nurs.

[11] Saleh ZT, Lennie TA, Alhurani AS, Almansour IM, Hamza A, Moser DK (2021). High dietary sodium intake is associated with shorter event-free survival in patients with heart failure and comorbid diabetes. Clin Nurs Res.

[12] Neily JB, Toto KH, Gardner EB, Rame JE, Yancy CW, Sheffield MA (2002). Potential contributing factors to noncompliance with dietary sodium restriction in patients with heart failure. Am Heart J.

[13] van der Wal MH, Jaarsma T, van Veldhuisen DJ (2005). Non-compliance in patients with heart failure; how can we manage it. Eur J Heart Fail.

[14] Creswell JW (2003). Research design: qualitative, quantitative, and mixed methods approaches.

[15] Graneheim UH, Lundman B (2004). Qualitative content analysis in nursing research: concepts, procedures and measures to achieve trustworthiness. Nurse Educ Today.

[16] Sabaté E (2003). Adherence to long-term therapies: evidence for action.

[17] Lennie TA, Moser DK, Chung ML (2020). Insight into differences in dietary sodium adherence between men and women with heart failure. J Cardiovasc Nurs..

[18] Smith JL, Lennie TA, Chung ML, Mudd-Martin G (2019). Dietary sodium intake is predicted by antihypertensive medication regimen in patients with heart failure. J Cardiovasc Nurs..

[19] Sattler ELP, Ishikawa Y, Trivedi-Kapoor R, Zhang D, Quyyumi AA, Dunbar SB (2019). Association between the prognostic nutritional index and dietary intake in community-dwelling older adults with heart failure: findings from NHANES III. Nutrients..

[20] Bidwell JT, Higgins MK, Reilly CM, Clark PC, Dunbar SB (2018). Shared heart failure knowledge and self-care outcomes in patient-caregiver dyads. Heart Lung..

[21] Wu JR, Lennie TA, Dunbar SB, Pressler SJ, Moser DK (2017). Does the theory of planned behavior predict dietary sodium intake in patients with heart failure. West J Nurs Res.

[22] Basuray A, Dolansky M, Josephson R (2015). Dietary sodium adherence is poor in chronic heart failure patients. J Card Fail.

[23] Chung ML, Lennie TA, Mudd-Martin G, Moser DK (2015). Adherence to a low-sodium diet in patients with heart failure is best when family members also follow the diet: a multicenter observational study. J cardiovasc nurs.

[24] Masterson Creber R, Topaz M, Lennie TA, Lee CS, Puzantian H, Riegel B (2014). Identifying predictors of high sodium excretion in patients with heart failure: a mixed effect analysis of longitudinal data. Eur J Cardiovasc Nurs.

[25] Masterson Creber R, Lee CS, Lennie TA, Topaz M, Riegel B (2014). Using growth mixture modeling to identify classes of sodium adherence in adults with heart failure. J Cardiovasc Nurs.

[26] Riegel B, Lee S, Hill J, Daus M, Baah FO, Wald JW, Knafl GJ (2019). Patterns of adherence to diuretics, dietary sodium and fluid intake recommendations in adults with heart failure. Heart Lung..

[27] Chung ML, Park L, Frazier SK, Lennie TA (2017). Long-term adherence to low-sodium diet in patients with heart failure. West J Nurs Res.

[28] Dolansky MA, Schaefer JT, Hawkins MA, Gunstad J, Basuray A, Redle JD (2016). The association between cognitive function and objective adherence to dietary sodium guidelines in patients with heart failure. Patient Prefer Adherence.

[29] Hwang SY, Kim J (2016). An examination of the association of cognitive functioning, adherence to sodium restriction and Na/K ratios in Korean heart failure patients. J Clin Nurs.

[30] Alosco ML, Spitznagel MB, van Dulmen M, Raz N, Cohen R, Sweet LH (2012). Cognitive function and treatment adherence in older adults with heart failure. Psychosom Med.

[31] Luyster FS, Hughes JW, Gunstad J (2009). Depression and anxiety symptoms are associated with reduced dietary adherence in heart failure patients treated with an implantable cardioverter defibrillator. J Cardiovasc Nurs..

[32] Lennie TA, Worrall-Carter L, Hammash M, Odom-Forren J, Roser LP, Smith CS (2008). Relationship of heart failure patients' knowledge, perceived barriers, and attitudes regarding low-sodium diet recommendations to adherence. Prog Cardiovasc Nurs.

[33] Chung ML, Moser DK, Lennie TA, Worrall-Carter L, Bentley B, Trupp R (2006). Gender differences in adherence to the sodium-restricted diet in patients with heart failure. J Card Fail.

[34] Evangelista LS, Berg J, Dracup K (2001). Relationship between psychosocial variables and compliance in patients with heart failure. Heart Lung.

[35] Mattes RD (1990). Discretionary salt and compliance with reduced sodium diet. Nutr Res.

[36] Ponikowski P, Voors AA, Anker SD, Bueno H, Cleland JG, Coats AJ (2016). 2016 ESC guidelines for the diagnosis and treatment of acute and chronic heart failure: the task force for the diagnosis and treatment of acute and chronic heart failure of the European Society of Cardiology (ESC). Developed with the special contribution of the heart failure association (HFA) of the ESC. Eur J Heart Fail.

[37] Lindenfeld J, Albert NM, Boehmer JP, Collins SP, Ezekowitz JA, Heart Failure Society of America (2010). HFSA 2010 comprehensive heart failure practice guideline. J Card Fail.

[38] Yancy CW, Jessup M, Bozkurt B, Butler J, Casey DE, Drazner MH (2013). 2013 ACCF/AHA guideline for the management of heart failure: a report of the American College of Cardiology Foundation/American Heart Association task force on practice guidelines. J Am Coll Cardiol.

[39] Kuehneman T, Saulsbury D, Splett PL (1998). Outcomes Management for the Patient with congestive heart failure. J Am Diet Assoc.

[40] Roura E, Foster S, Winklebach A, Navarro M, Thomas W, Campbell K, Stowasser M (2016). Taste and hypertension in humans: targeting cardiovascular disease. Curr Pharm Des.

[41] Elliott P, Brown I. Sodium intakes around the world: background document prepared for the forum and technical meeting on reducing salt intake in populations. World health. Organization. 2006; http://www.who.int/dietPhysical activity/Elliot -brown-2007.pdf.

